# Alpha lipoic acid attenuates radiation-induced oral mucositis in rats

**DOI:** 10.18632/oncotarget.20286

**Published:** 2017-08-16

**Authors:** Jin Hyun Kim, Myeong Hee Jung, Jin Pyeong Kim, Hyun-Jung Kim, Jung Hwa Jung, Jong Ryeal Hahm, Ki Mun Kang, Bae-Kwon Jeong, Seung Hoon Woo

**Affiliations:** ^1^ Biomedical Research Institute, Gyeongsang National University Hospital, Jinju, Gyeongnam, Republic of Korea; ^2^ Institute of Health Science, Gyeongsang National University, Jinju, Gyeongnam, Republic of Korea; ^3^ Department of Otolaryngology, Gyeongsang National University, Jinju, Gyeongnam, Republic of Korea; ^4^ Department of Internal Medicine, Gyeongsang National University, Jinju, Gyeongnam, Republic of Korea; ^5^ Department of Radiation Oncology, Gyeongsang National University, Jinju, Gyeongnam, Republic of Korea; ^6^ Beckman Laser Institute, University of California, Irvine, California, USA

**Keywords:** alpha lipoic acid, oral mucosa, radiation, Hif-1a, complications

## Abstract

**Purpose:**

Radiotherapy is currently one of the main treatment modalities for head and neck cancer; however, it also results in severe toxicity to the normal tissue, to the detriment of patients. This study aimed to investigate whether alpha lipoic acid (ALA) could protect against radiation-induced oral mucositis in a rat model.

**Results:**

On post-irradiation days 4 and 7, the epithelial layer on oral mucosa showed pronounced injury (shortening of the layer) and it is diminished by ALA pretreatment before radiation. Hif-1a expression was significantly induced in the radiation group on days 4, 7, and 28. GLUT1 expression was also induced by radiation at all time points, and the expression levels peaked on day 28. Phosphorylated p53 level was significantly higher in the radiation group on days 4 and 7, and Bax protein expression was significantly higher in the same group on day 4 than ALA-pretreated radiation group. TUNEL-positive staining was significantly lower in the ALA-pretreated radiation group.

**Materials and methods:**

Rats were assigned to one of the following four groups: control, ALA only (100 mg/kg, i.p.), irradiated, and ALA administered 24 h and 30 min prior to irradiation, with the neck area including the oral mucosa evenly irradiated with 2 Gy per minute (total dose, 18 Gy) using a photon 6-MV linear accelerator. Rats were sacrificed 4, 7, 28, or 56 days after radiation.

**Conclusions:**

The results show that ALA can be used to ameliorate radiation-induced oral mucositis with head and neck cancer.

## INTRODUCTION

Radiation therapy is the primary treatment for patients with head and neck cancer, but it leads to the development of oral mucositis accompanied by tissue structure destruction and functional alteration. It should be noted that managing oral mucositis should be based on the pathobiology of the radiation-induced oral mucositis [1]. Among other symptoms, dry mouth and loss of taste can also occur during radiotherapy and occasionally persist for a long time after the therapy concludes [2]. It is known that irradiated oral mucosa, even after the complete healing of mucositis, become atrophic and susceptible to injury. Although we are responsible for managing the oral mucosa after radiotherapy, there is little information on the molecular basis for this vulnerability of irradiated oral mucosa.

In the initiation stage of radiation-induced oral mucositis, DNA damage and reactive oxygen species (ROS) release activate apoptotic factors such as p53 and the Bcl-2 family in the affected cells [3, 4]. Recently, Hif-1a seems to be a key transcription factor in activating apoptotic cell death in mucositis, [1, 5] and if Hif-1a plays a central role in the development of radiation-induced mucositis, it may also be implicated in the vulnerability of normal oral mucosa that are exposed to radiotherapy. However, only few reports have studied Hif-1a expression in oral mucosa after radiotherapy, with one report by Gruber et al. describing that Hif-1a played a critical role in the occurrence of radiation-induced oral mucositis [1, 6].

Recently, it has been reported that alpha lipoic acid (ALA) protects against radiation-induced normal tissue injury and dysfunction [7–9]. ALA is a strong antioxidant with high reactivity to free radicals, and it elevates tissue levels of glutathione [10]. ALA has been demonstrated to be effective in preventing pathological processes such as ischemia-reperfusion injury [11], diabetes [12], hypertension, and radiation injury [13].

In the present study, we aimed to investigate the protective effects of ALA on radiation-induced oral mucositis. We assumed that Hif-1a expression would be positively correlated with functional and structural tissue injury and apoptosis by radiation and that ALA would mitigate this injury.

## RESULTS

### ALA protected against radiation-induced oral mucosa injury

**Figure 1:**
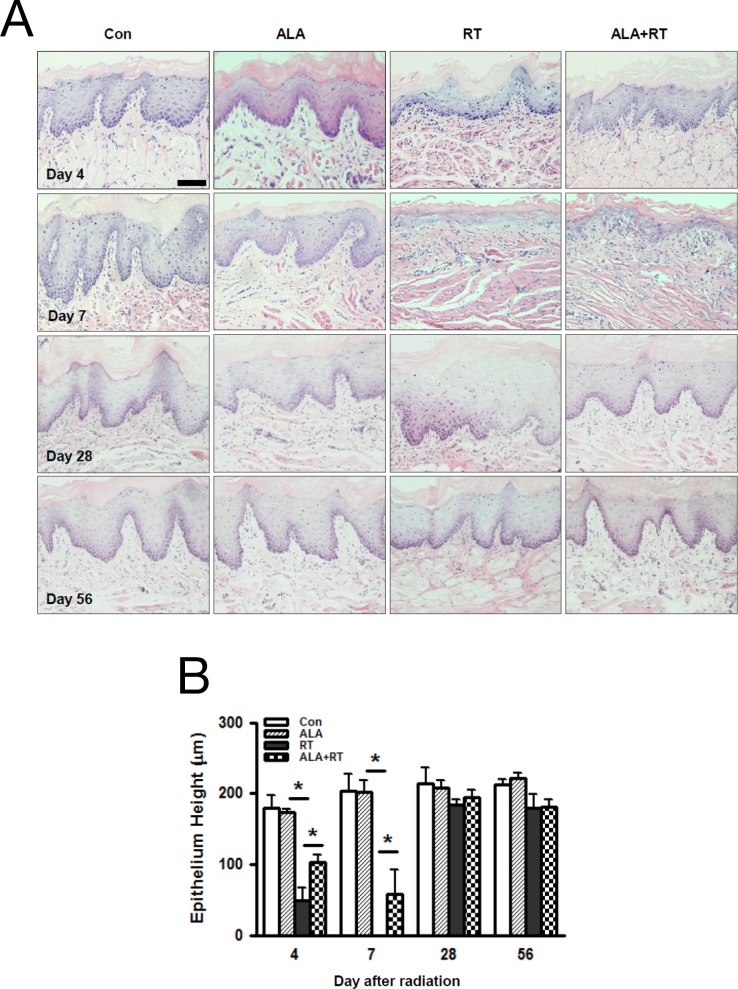
Histopathological changes in the oral mucosa 4, 7, 28, and 56 days after irradiation ALA decreases pathological changes in the oral mucosa after irradiation. All tissues were sectioned and stained with H&E **(A)**. Exposing the head and neck area to irradiation resulted in a shortened squamous epithelium, but this was decreased in ALA+RT **(B)**. Con, control; ALA, ALA only; RT, irradiated; ALA+RT, ALA before irradiation. Scale bar, 100 μm.

Figure 1A shows the typical damage to the mucosa with destruction of the squamous epithelium, the atrophic changes in the epithelium, the sterilization of mucosal stem cells, and the resulting edematous cellular morphology in the RT group, whereas the same cell types were less frequent in the ALA+RT group. We evaluated the histological changes by measuring the squamous epithelium height in the oral mucosa. At all time points, we found no significant differences between CON and ALA, but the height decreased significantly in RT compared with CON on days 4 and 7 (CON *vs*. RT, day 4: 180.127 μm *vs*. 49.088 μm; day 7: 203.277 μm *vs*. 0 μm, *p* < 0.05; Figure 1B). In particular, radiation resulted in a loss of the entire thickness of the epithelium with a small quantity of muscle tissue on day 7. The height decrease in ALA+RT was significantly lower than the decrease in RT on days 4 and 7 (RT *vs*. ALA+RT, day 4: 49.088 μm *vs*. 103.58 μm; day 7: 0 μm *vs*. 58.286 μm, *p* < 0.05; Figure 1B). On day 28 and 56, there is no significant difference between ALA+RT and RT groups.

### ALA reduced Hif-1a and GLUT1 expression in the germinal and epithelial layers

**Figure 2:**
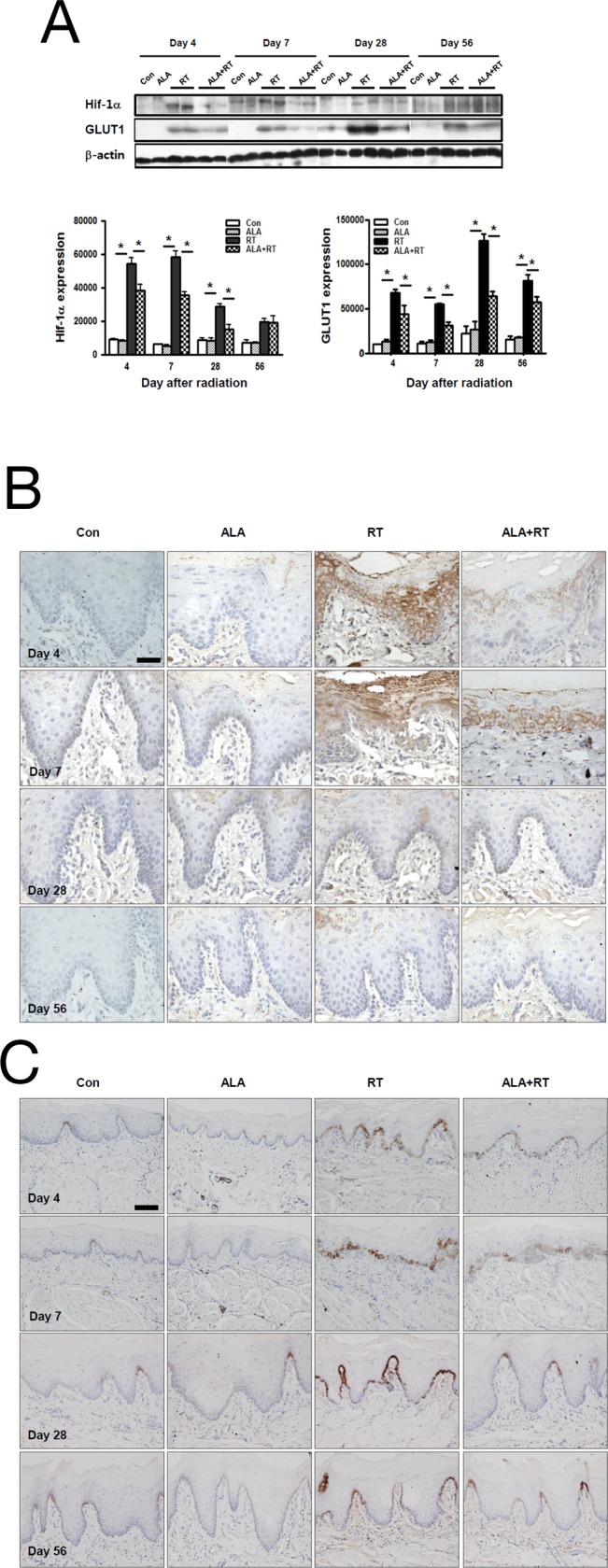
ALA decreases Hif-1a and GLUT1 expression in the oral mucosa Western blotting was performed with anti-Hif-1a and anti-GLUT1 antibodies. Hif-1a and GLUT1 expression were induced in RT at days 4, 7, and 28 after irradiation, and this expression occurred on days 7, 28, and 56 in CON and ALA+RT. B-actin was used as the loading control **(A)**. Strong Hif-1a–positive signals were detected in RT on days 4 and 7, and ALA pretreatment decreased the signals **(B)**. GLUT1-positive signals were detected in RT on all days, and ALA pretreatment decreased the signals **(C)**. Con, control; ALA, ALA only; RT, irradiated; ALA+RT, ALA before irradiation. Scale bar, 50 μm for Hif-1a and 100 μm for GLUT1.

Our results showed that Hif-1a expression was significantly increased in RT on days 4, 7, and 28, but it decreased significantly with ALA pretreatment; we found no significant differences between RT and ALA+RT on day 56. We did observe positive signals for Hif-1a in the germinal and epithelial layers of the squamous epithelium, and the signal was particularly abundant on day 4; on day 7, the expression was mainly in the germinal layer in ALA+RT (Figure 2B). Similar to Hif-1a, radiation also induced GLUT1 expression at all time points, and the expression levels peaked for the samples from day 28 in RT. ALA pretreatment significantly reduced expression levels on days 7, 28, and 56 (Figure 2A). GLUT1 levels clearly showed membrane-associated expression in the germinal layer of the squamous epithelium in RT at all time points. In particular, GLUT1 expression gradually increased in RT, peaked on day 28, and decreased on day 56, and ALA pretreatment attenuated this GLUT1 expression increase on days 4, 7, and 28; however, it was not significant on day 56 (Figure 2C).

### ALA reduced protein expression of pro-apoptotic factors in the oral mucosa after radiation

**Figure 3:**
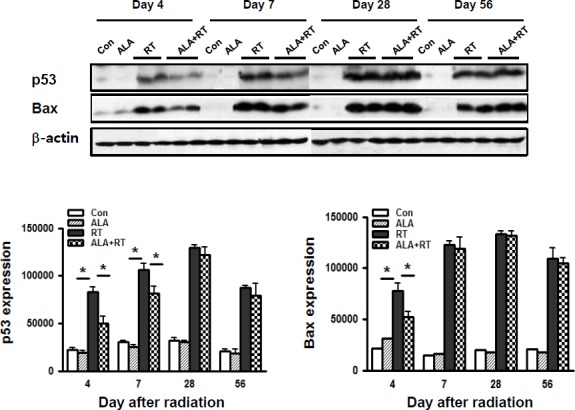
ALA decreased radiation-induced apoptotic signals in the oral mucosa Western blotting was performed using anti-p-p53 and Bax. In RT, p53 expression was induced on days 4 and 7, and Bax expression was induced by day 4. β-actin was used as the loading control. Con, control; ALA, ALA only; RT, irradiated; ALA+RT, ALA before irradiation.

We also investigated the apoptotic activity in the oral mucosa. Figure 3 shows that phosphorylated p53 and Bax protein expression were significantly increased in the radiation group on days 4 and 7 and day 4, respectively, but these expression increases were significantly dampened by ALA pretreatment. Phosphorylated p53 level showed no significant differences on day 28 and 56 between ALA+RT and RT groups. In contrast, ALA pretreatment significantly reduced Bax protein expression only on day 4 after the radiation treatment. We observed only negligible signals for phosphorylated p53 and Bax in CON and ALA at all time points.

### ALA decreased apoptosis of the squamous epithelium in the oral mucosa

**Figure 4:**
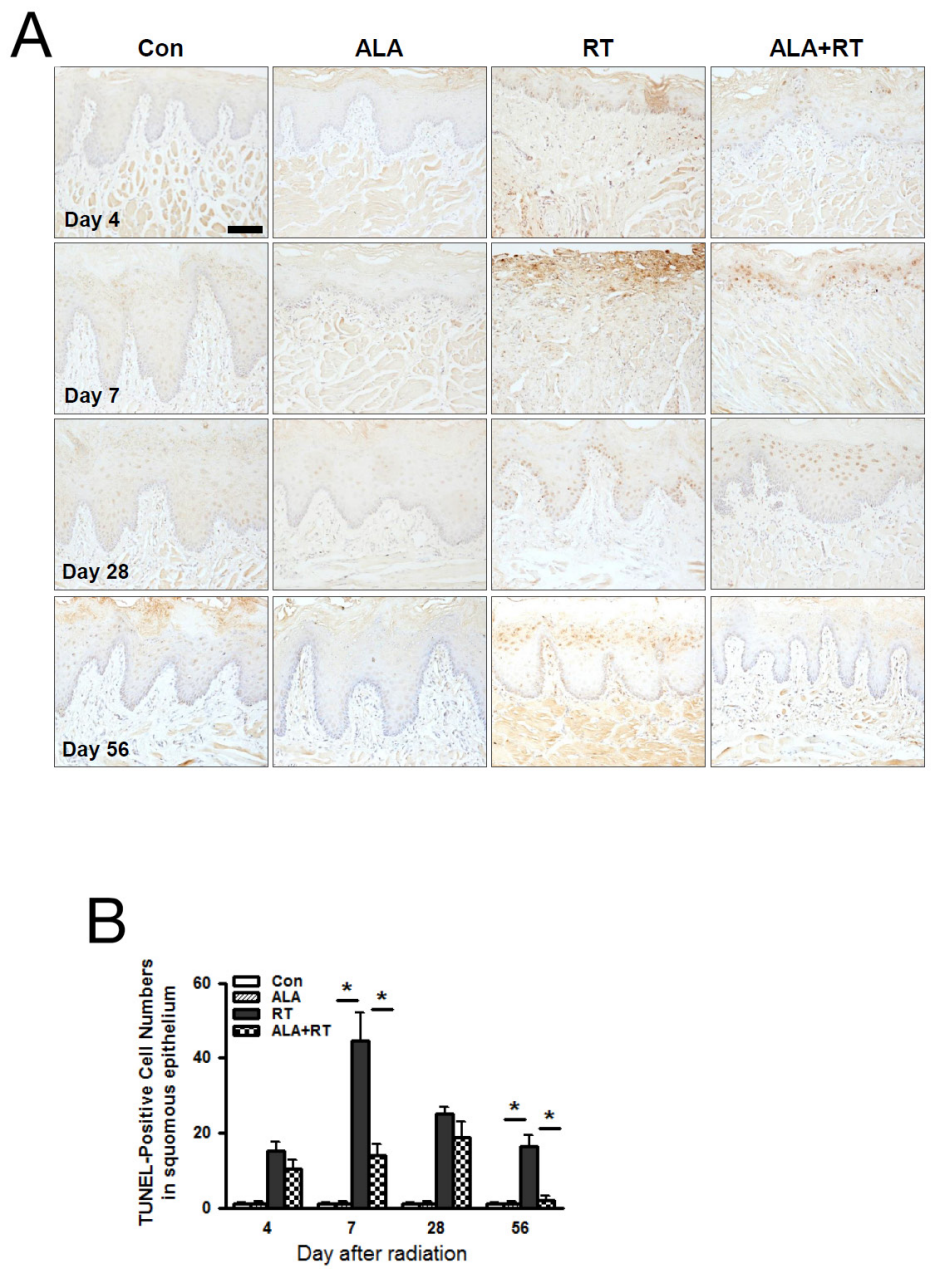
ALA decreased radiation-induced cell death in the squamous epithelium of the oral mucosa Cell death was analyzed with the TUNEL assay **(A)**. Microscopic image shows increased cell death in RT compared with Con and ALA+RT at 4 and 7 days after irradiation **(B)**. Scale bar, 100 μm.

Apoptosis is one of the major pathogenic aspects of mucositis, and the degree of apoptosis reflects the degree of mucositis. We assessed apoptosis levels using TUNEL staining, and at all time points, we found no significant differences between CON and ALA; we detected most of the positive signals at the germinal layer of the squamous epithelium and some at the functional epithelial layer (Figure 4A). In RT, we detected the TUNEL-positive signal in the new germinal layer on day 28, and this positive signal moved to the epithelial layer on day 56. In ALA+RT, we detected the TUNEL-positive signal in the germinal layer on day 7, and this signal also moved to the epithelium on day 28, followed by nearly disappearing on day 56 (Figure 4A). These TUNEL-positive signals increased significantly in RT at all time points; notably, the image from RT on day 7 shows that the squamous epithelium was appreciably injured by the radiation. ALA+RT showed a significant reduction in TUNEL-positive signals on day 7 compared to RT (Figure 4B).

## DISCUSSION

Apart from the targeted cancer cells, highly proliferative normal cells are the most affected by radiation therapy. The oral mucosa are particularly vulnerable to radiation because the mucosa consists of cells with highly self-renewing activity, and with this high turnover rate, the lining replaces itself every 7 to 14 days. Oral mucositis by radiation is an acute and transient adverse effect, and its onset from radiotherapy is a biologically complex process [17, 18]. Although radiation-induced oral mucositis spontaneously resolves 3–4 weeks after treatment, oral mucosa that are exposed to radiation appear to become atrophic and susceptible to injury even after they are completely re-epithelialized [18]. Cells in the oral mucosa that are harmed by radiation include squamous epithelial cells and submucosal stromal cells, which comprise fibroblasts and vascular endothelial cells. Radiation and radiation-induced ROS generation damage the DNA in the cell itself, lead to apoptosis or necrosis of squamous epithelial cells, and inhibit cell proliferation, which triggers the breakdown of the squamous cell layer [17, 18].

In the acute phase after radiation, inflammation by cytokines released from the basal epithelium and/or the adjacent connective tissue causes increased subepithelial vascularity and reduced epithelium thickness. These transformations are known to result in signs of local hypoxia [1, 6]. When cells are exposed to extreme or prolonged hypoxia such as with irradiation, Hif-1a does not protect cells from the adaptive mechanisms, but the cells can face Hif-1a-involved apoptosis [19]. Hypoxic conditions can induce apoptosis, but the role of Hif-1a in this process is still controversial; some studies reported that Hif-1a mediates hypoxia-induced cell death by activating the transcription of many pro-apoptotic genes such as *NIX* and *NIP3*, part of the pro-apoptotic Bcl-2 family [20, 21], and others report that Hif-1a-dependent p53 could mediate the induction of apoptosis in hypoxia [19, 22]. It was also shown that hypoxia could stabilize p53, including increased p53 phosphorylation [23–25]. Researchers have also detected a direct association between Hif-1a and p53 under hypoxic conditions [24, 26]. In hypoxia, p53 is stabilized, and p53 is a significant transcription factor in activating cell death-initiating genes including Bax and in arresting p21 growth in response to DNA damage or stress [21, 23, 25]. The findings from these previous studies are similar to our results. As shown in Figures 2A, 2B, and 3, increased Hif-1a expression correlated highly with increased p53 phosphorylation and Bax expression in RT on days 4 and 7 after radiation. As noted above, p53 mediated apoptosis in RT might be dependent on Hif-1 alpha. Our results indicate that head and neck radiotherapy leads to hypoxia of the oral mucosa, and this hypoxia increases p53 stabilization by increasing phosphorylated p53 levels, leading to increased pro-apoptotic Bax expression, and, finally, oral mucositis. However, ALA might be involved in ameliorating these expression levels in the acute phase. This result suggests that ALA might be involved in Hif-1a-p53-dependent apoptotic epithelial cell death in the oral mucosa after radiation. However, most studies have shown that elevated Hif-1a expression in tumor cells confers resistance to hypoxic exposure that ultimately contributes to tumor resistance to radiotherapy and chemotherapy [20–22]. Because HIF-1a plays an important role in protecting solid tumors against hypoxia by promoting angiogenesis, inducing growth factor expression, preventing apoptosis, and increasing anaerobic metabolism [27, 28]. In particular, HIF-1a can confer resistance to apoptosis under hypoxia by increasing GLUT-1 [29]. Interestingly, this coincides with our findings shown in Figures 2A and 2C. As mentioned above, radiation significantly increased Hif-1a expression, but ALA pretreatment reduced the expression. Radiation also significantly increased GLUT-1 expression, and ALA also reduced this expression at all time points. Excess radiation exposure can reduce the blood and nutrient supply to abnormal blood vessels in the submucosa, which can lead to epithelial cell death and damage to the basal layer. Most cells have defense mechanisms to protect themselves against insults, and the increased Hif-1a and GLUT-1 expression could be one of these mechanisms for protecting against radiation insult. Hif-1a-GLUT-1-dependent pathway might be a defense response to overcome Hif-1a-p53-dependent apoptotic epithelial cell death. However, ALA pretreatment might create a less hypoxic environment, which could lead to less Hif-1a-p53-dependent apoptotic machinery and less Hif-1a-GLUT-1-dependent adaptation.

Interestingly, ALA might be related to epithelial and basal layer regeneration. The TUNEL assay in Figure 4 shows increased cell death and a shortened epithelial layer in RT and ALA+RT compared with CON in the early period (days 4 and 7). However, in ALA+RT, the TUNEL-positive cells were detected only at the basal layer on day 7; these cells were moving toward the outer layer on day 28, and they had disappeared by day 56. This cell movement could have been caused by the regeneration of the epithelial layer. When the oral mucosa were exposed to radiation, the epithelial layer including the basal layer was destroyed, but the basal layer regenerated and the epithelial layer was generated from the basal layer; that is, the TUNEL-positive cells moved toward the outer layer over time. Thus, ALA+RT showed more advanced repair of the basal and epithelial layers than did RT, which suggests that ALA could be involved in the oral epithelial regeneration after radiation.

There were a few limitations to this study. First, the single dose of 18Gy that we used is not relevant for treating head and neck cancer in the clinic; because the doses we give patients are limited by the response of normal tissue, fractionated doses are more common and more beneficial in treatment. In addition, we were focused on the protective effect of ALA against radiation-induced oral injury, and thus, it was necessary to give the rats the high dose. Second, we should have verified that ALA did not promote cancer growth or prevent radiation-induced cancer cell death. Although studies have reported on ALA and cancer cell growth and death, the role of ALA in cancer cells is still controversial. We designed this work as a feasibility study of cancer cell growth and death and ALA treatment.

In conclusion, our results show that ALA could be used to ameliorate radiation-induced oral mucositis with head and neck cancer.

## MATERIALS AND METHODS

### Ethics statement

The Gyeongsang National University Institutional Animal Care & ethics committee specifically approved this study (GLA-120120-R0002).

### Radiation exposure

We assigned male Sprague–Dawley rats (230–250 g; Koatech Inc., Peongtaek, Korea) to the following groups: control, n=12 (CON); irradiated, n=16 (RT); ALA administered before irradiation, n=16 (ALA+RT); and ALA administered alone, n=12 (ALA). We administered the ALA (100 mg/kg, i.p., Bukwang Pharmaceutical Co., Seoul, Korea) 24 h and 30 min before irradiation, and we chose the dose and frequency based on previous studies [14–16]. The neck area was evenly irradiated with 2 Gy/min (total dose, 18 Gy) using a photon 6-MV linear accelerator (21EX, Varian, Palo Alto, CA, USA). A 3-cm block of Lucite was positioned above the head and neck to provide adequate buildup and facilitate even radiation distribution. Each rat was exposed to a single dose of radiation and sacrificed 4, 7, 28, or 56 days after radiation.

### Histopathology: height of the squamous epithelium

Tissues were fixed in 4% paraformaldehyde in 0.1 M PBS, embedded in paraffin, and cut into 5-μm sections. The sections were stained with hematoxylin and eosin (H&E) and Masson's trichrome (Masson's Trichrome Kit, Sigma Diagnostics, St. Louis, MO, USA). We measured the squamous epithelium height (from the top of the epithelium to the germinal layer) under light microscopy (×200) using a calibrated micrometer and NIS Elements BR3.2 software (Nikon, Tokyo, Japan). We measured and averaged 10 intact oral (especially buccal area) mucosa sections with squamous epithelium samples per rat for each sample.

### TUNEL assay

We assessed the degree of apoptosis using the TUNEL assay (Roche, Indianapolis, IN, USA), conducting semi-quantitative analysis by counting the TUNEL-positive cells per field at 400× magnification; we randomly selected at least 10 areas in the squamous epithelium of each oral mucosa per slide for analysis, and we considered the mean number of brown cells in the selected fields to be the number of TUNEL-positive cells. A blind observer analyzed the signals in the 10 randomly selected fields using NIS Elements BR3.2.

### Immunoblotting

The tissues were homogenized in a lysis buffer. Fifty micrograms of proteins were loaded on a sodium dodecyl sulfate-polyacrylamide gel. The blots were probed with primary antibodies to polyclonal anti-hypoxic inducible factor-1 alpha (Hif-1a (diluted 1:1000; ab2185, Abcam, Cambridge, MA, USA), anti-phosphorylated p53 (diluted 1:500; #9284, Cell Signaling, Danvers, MA, USA), anti-Bax (diluted 1:500; #2772, Cell Signaling), and monoclonal anti-glucosetransporter (GLUT1; diluted 1:1000; ab40084, Abcam) at 4°C overnight. The primary antibody was visualized using secondary antibodies with an enhanced chemiluminescence kit (Amersham Pharmacia Biotech, Piscataway, NJ, USA).

### Immunohistochemistry

After deparaffinization, the sections were incubated with primary antibodies to polyclonal anti-Hif-1a (diluted 1:100; ab2185, Abcam), monoclonal anti-GLUT1 (diluted 1:200; ab40084, Abcam), monoclonal anti-Ki67 (diluted 1:50; ab16667, Abcam), monoclonal anti-PCNA (diluted 1:200; sc-56, Santa Cruz Biotechnology, Dallas, TX, USA), and monoclonal anti-8-hydroxydeoxyguanosine (diluted 1:500; ab62623, Abcam); with biotin-conjugated secondary IgG (diluted 1:200; Vector Laboratories, Burlingame, CA, USA); with the avidin-biotin-peroxidase complex (ABC Elite Kit, Vector Laboratories); and with diaminobenzidine tetrahydrochloride. We then visualized the sections under light microscopy and captured and analyzed the digital images.

### Statistical analysis

We conducted all statistical analyses using SPSS (SPSS Inc., Chicago, IL, USA), and results are presented as mean ± standard error of the mean. We assessed the differences between groups using one-way analysis of variance followed by the Student–Newman–Keuls test, and we considered a P-value of less than 0.05 significant.
